# The impact of horizontal violence among nurses on their job burnout: a moderated mediation model

**DOI:** 10.3389/fpubh.2025.1633956

**Published:** 2025-09-24

**Authors:** XuYan Liu, RenLong Liang, YiWei Li, Yin Yuan, Tingting Ruan, Rui Jian

**Affiliations:** ^1^Department of Gynecology, Deyang People’s Hospital, Deyang, Sichuan, China; ^2^Department of Neurology, Deyang Hospital Affiliated Hospital of Chengdu University of Traditional Chinese Medicine, Deyang, Sichuan, China

**Keywords:** nurses, horizontal violence, job burnout, professional mission, psychological detachment, moderated mediation

## Abstract

**Objective:**

Chinese nurses have a heavy workload, and the problem of inter nurse horizontal violence is prominent. Nurses who are subjected to horizontal violence are more likely to experience professional burnout. The aim of this study is to explore the intrinsic relationship between inter nurse horizontal violence and nurse burnout, and to examine the mediating role of psychological detachment in this relationship. In addition, this study also evaluated whether professional mission has a moderating effect in this mediating model.

**Methods:**

From November to December 2024, this study employed a cross-sectional survey method to recruit nurses from five tertiary first-class public hospitals in the southwest region of Sichuan Province. Surveys were conducted using general information questionnaires, lateral violence among nurses questionnaires, job burnout scales, psychological detachment scales, and professional mission scales. Data obtained from the survey were analyzed using SPSS 27.0 and its macro program Process v4.2.

**Results:**

Research indicates that inter-nurse horizontal violence is a significant predictor of job burnout, with a positive correlation between the two. Psychological detachment has been identified as playing a partial mediating role in the association between inter-nurse horizontal violence and job burnout, with the mediating effect accounting for 44.73% of the total effect. Furthermore, a sense of professional mission weakens the negative prediction of horizontal violence on psychological detachment and the positive prediction of horizontal violence on job burnout, and moderates the first half of the mediating effect model as well as the direct effect.

**Conclusion:**

Nurses’ psychological detachment ability plays a partial mediating role in horizontal violence and job burnout, with a sense of professional mission moderating this mediation model. Nurses with a strong sense of professional mission are more likely to overcome the distress caused by horizontal violence, enabling them to have a higher degree of psychological detachment, recover during rest, and thus reduce job burnout. Conversely, nurses with a weak sense of professional mission are more prone to experiencing job burnout when subjected to horizontal violence. Therefore, enhancing nurses’ sense of professional mission and psychological detachment ability is beneficial for alleviating job burnout among nurses.

## 1 Introduction

Job burnout refers to a state of extreme physical and emotional fatigue experienced by employees in the face of persistent sources of work pressure, including despair, fatigue, anger, and negative attitudes (1). Maslach et al. (2) further developed the theory of job burnout, positing that it encompasses the following three aspects: first, emotional exhaustion, manifested as emotional depletion and a lack of emotional resources; second, depersonalization, characterized by negative reactions towards others and a loss of idealism; third, reduced personal accomplishment, reflected in a decline in work ability and self-expression.

China has a large population, and the aging population problem is severe. Chinese nurses face a high intensity of clinical work, and due to frequent night shifts, their schedules are irregular, leading to severe sleep disorders in many nurses (3). Additionally, with the increasing demand for patient health and the rising awareness of rights protection, the doctor-patient conflict remains prominent (4). The combination of these factors has made the nurse population a high-risk group for job burnout. A cross-sectional study involving 25,120 medical staff in China showed that 60.8% of participants reported at least one symptom of job burnout, while 11.2% reported three symptoms of job burnout (5), indicating that the current situation of job burnout among nurses is not optimistic. Further research (6) shows that job burnout among nurses not only affects their physical and mental health but also further endangers patient safety and affects the quality of nursing services. Therefore, exploring the factors influencing job burnout among nurses is of great significance for alleviating burnout, maintaining the stability of the nursing team, and ensuring patient safety.

Due to prolonged high-intensity work, lateral violence is more likely to occur among nurses. Lateral violence among nurses refers to hostile behaviors within the nursing profession that are characterized by aggression, bullying, intimidation, or divisiveness. Lateral violence can manifest as overt behaviors such as belittling, slandering, and spreading rumors, as well as more subtle and covert behaviors such as concealing information, isolating, snubbing, and undermining promotion and learning opportunities (7, 8). Studies have shown (9) that lateral violence is prevalent among nurses, with the incidence of lateral violence among nurses ranging from 7 to 83% across different regions, which varies significantly due to cultural differences and different work environments in different regions (10). Lateral violence not only leads to subjective feelings such as fatigue, insomnia, stress, and humiliation among nurses, harming their physical and mental health, but also reduces their willingness to collaborate and exacerbates job burnout (11), thereby adversely affecting the quality of nursing services, patient safety, and the development of the nursing workforce.

### 1.1 The relationship between horizontal violence among nurses and job burnout

The Conservation of Resources (COR) theory posits that individuals are motivated to strive to acquire, maintain, cultivate, and protect resources in order to adapt to the environment and sustain their basic survival needs (12). According to the COR theory (13), individuals with depleted resources are more sensitive to and vulnerable to resource loss, and thus they may adopt defensive strategies (such as reducing resource investment in other activities) to avoid further resource loss (14). Inter-nurse horizontal violence can deplete nurses’ emotional and psychological resources, leading to emotional exhaustion (a core dimension of job burnout), a lack of psychological resources, and a self-protection mechanism that causes nurses to reduce their resource investment in their work, ultimately triggering job burnout (14).

The Job Demand-Resources Model (JD-R) provides further theoretical support (15). The JD-R model posits that job burnout is caused by high job demands and insufficient job resources. When nurses face horizontal violence, they cannot receive respect and support from colleagues, leading to a decline in job resources at the organizational level. Furthermore, horizontal violence can also cause depletion of nurses’ “emotional resources,” leading to an imbalance between job demands and job resources, ultimately resulting in job burnout.

Previous research also supports this result. A cross-sectional study involving 1,761 Chinese nurses revealed (16) that there is a significant positive correlation between horizontal violence and job burnout, indicating that nurses who experience horizontal violence are more prone to developing job burnout. Therefore, horizontal violence can serve as a predictor of job burnout. Based on the above theory, a hypothesis is proposed.

*Hypothesis 1*: There is a significant positive correlation between inter-nurse horizontal violence and job burnout among nurses.

### 1.2 The mediating role of psychological detachment

Psychological detachment, proposed by Etzion (17) in 1998, refers to the feeling of individuals being away from the work environment. Sonnentag et al. (18) further enriched the concept of psychological detachment, which refers to employees actively and proactively distancing themselves from work during non-working hours after getting off work, no longer thinking about work-related matters, not being occupied by work, and being able to freely manage their time. However, driven by modern information and communication technology, the boundaries between work and life are becoming increasingly blurred. The widespread use of smart electronic devices enables hospital leaders to contact medical staff and assign work tasks at any time, and it has become normal for medical staff to handle work tasks outside working hours (19). As an effective self-regulation method, active psychological detachment not only helps nurses recover the “emotional resources” consumed by work, but also enables nurses to increase their work engagement during working hours, better complete their work, and reduce the occurrence of job burnout (20).

The Effort-Recovery Theory posits that individuals need to recover physiological and psychological resources through behaviors such as rest and stress management after sustained high-load work, in order to prevent the negative impact of long-term resource depletion on job engagement (21). Psychological detachment is a necessary condition for resource recovery, and active psychological detachment is also beneficial for the recovery of personal resources (22). That is, nurses should not be dominated by work during non-working hours, not recall their own work, and invest their time in areas they are interested in, which will help nurses recover their personal resources. According to the COR (15) theory, personal resource recovery is conducive to nurses investing more resources into their work, thereby reducing job burnout. Therefore, how to improve nurses’ psychological detachment ability, enabling them to detach from heavy clinical and scientific research tasks during their rest time, and thus allowing nurses to recover both physically and psychologically, remains a problem that requires attention.

Inter-nurse horizontal violence is also closely related to psychological detachment. When nurses experience horizontal violence, it can lead them to repeatedly recall the conflict during non-working hours, continuously activating work pressure and hindering the recovery of psychological resources (23). Therefore, inter-nurse horizontal violence often leads to a decrease in nurses’ ability to detach psychologically. Conversely, reducing inter-nurse horizontal violence can enhance nurses’ ability to detach psychologically, allowing them to recover more psychological resources during rest, thereby engaging more actively in work and reducing the occurrence of job burnout.

Psychological detachment is an effective way to alleviate job burnout and its negative consequences. A cross-sectional study involving 1,861 nurses working 12-h shifts revealed (24) that when nurses are able to actively detach from their work during breaks, their job engagement is higher, and job burnout is less likely to occur. Additionally, Cropley et al.’s (25) research indicates that workplace horizontal violence can weaken employees’ psychological detachment by triggering rumination, thereby negatively affecting their mental health. That is, horizontal violence may lead to emotional resource depletion and trigger job burnout by weakening psychological detachment ability. Based on the above theoretical and empirical research findings, the following hypothesis is proposed:

*Hypothesis 2*: There is a negative correlation between inter-nurse horizontal violence and nurses’ psychological detachment.

*Hypothesis 3*: There is a negative correlation between nurses’ psychological detachment and job burnout.

*Hypothesis 4*: Nurses’ psychological detachment plays a mediating role in inter-nurse horizontal violence and job burnout.

### 1.3 The moderating effect of professional mission

Professional mission refers to an individual’s strong passion for their occupation, which can generate a profound sense of meaning and responsibility in their work (26). Nursing is a profession dedicated to saving lives and healing the wounded, a job that benefits others and society, thus inherently possessing a noble quality. Research indicates that the overall professional mission of Chinese nurses is at a medium or above-medium level (27), indicating significant room for improvement. Enhancing nurses’ professional mission can help strengthen their understanding of the significance and responsibility of their work, ultimately leading to improved nursing quality and better patient health outcomes.

The Meaning Maintenance Model (MMT) (28) posits that humans inherently seek to extract meaning from their environment and rely on stable meaning frameworks (such as a sense of professional mission) to understand the world. When individuals encounter events that conflict with their existing meaning frameworks (such as horizontal violence), a “meaning crisis” is triggered, manifesting as anxiety, confusion, or unease. When the existing meaning framework is threatened, individuals will re-establish psychological balance through various compensatory mechanisms (29).

Among nurses, when experiencing horizontal violence, their original meaning framework is disrupted. Nurses perceive the horizontal violence they endure as a test of their professional ideals, and they mitigate its negative effects through cognitive restructuring (30, 31). Specifically, nurses with a strong sense of professional mission, by endowing their work with a higher meaning, can buffer the impact of horizontal violence on psychological detachment when confronted with it. Additionally, nurses with a strong sense of professional mission exhibit greater psychological resilience, which can reduce the occurrence of job burnout. Conversely, nurses with a weak sense of professional mission are more prone to experiencing negative emotions when facing horizontal violence, leading to psychological detachment, decreased psychological endurance, and an increased likelihood of job burnout.

Liu et al. (32) conducted a cross-sectional study involving 744 operating room nurses from 12 hospitals in Shandong Province, China. Their findings indicated that a higher sense of professional mission was associated with lower levels of job burnout. Based on the aforementioned theoretical and empirical research results, we reasonably infer that a strong sense of professional mission weakens the negative predictive effect of horizontal violence on psychological detachment and simultaneously diminishes its positive predictive effect on job burnout. We further propose the following hypothesis.

*Hypothesis 5*: There is a positive correlation between professional mission and psychological detachment among nurses.

*Hypothesis 6*: There is a negative correlation between professional mission and job burnout among nurses.

*Hypothesis 7*: Professional mission moderates the first half of the mediation effect model as well as the direct effect.

### 1.4 Construction of the regulation model

**Figure 1:**
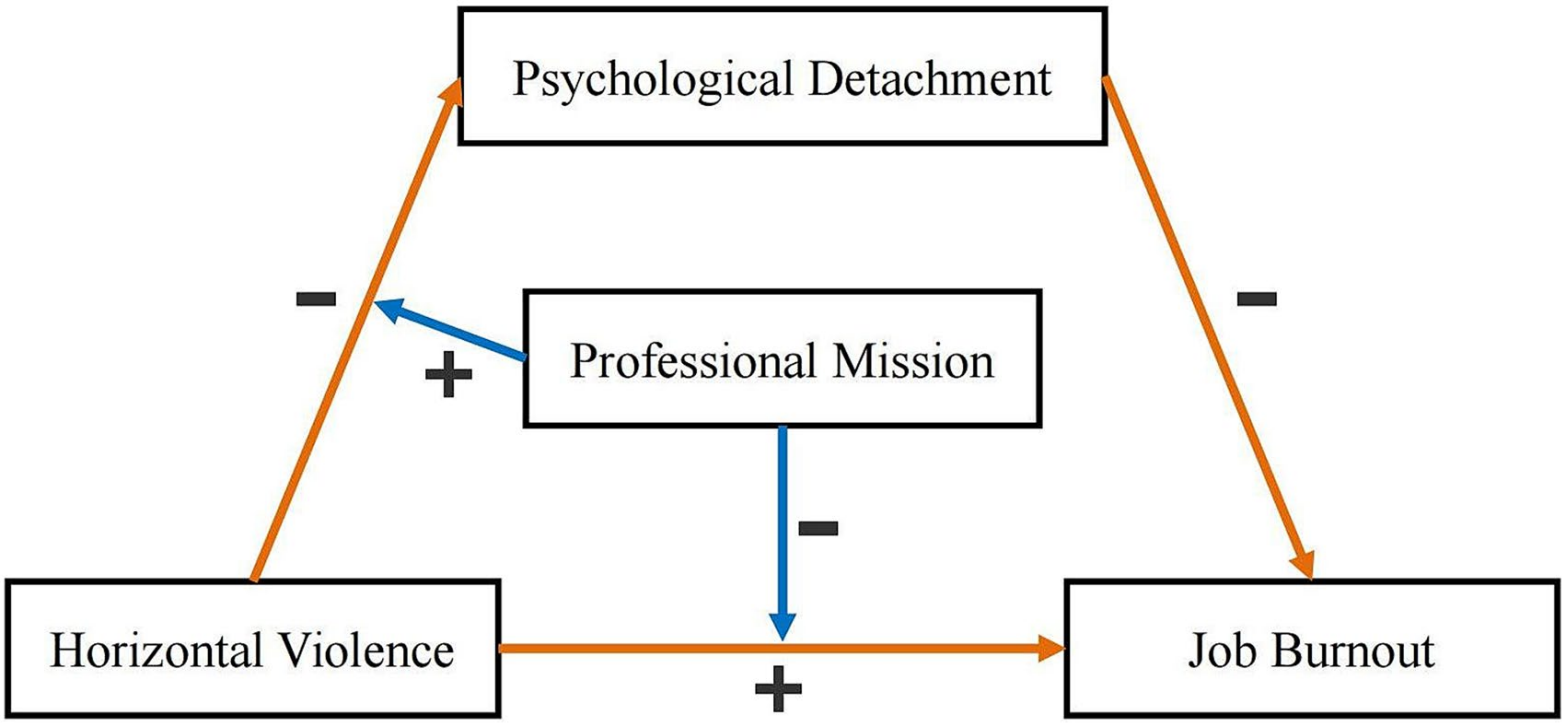
Hypothesis-based structural equation diagram.

Based on the aforementioned theories and hypotheses, this study constructed a moderated effect model (see Figure 1). The purpose of this study is to verify the hypotheses mentioned above and explore the complex intrinsic relationship between inter-nurse horizontal violence and job burnout. It is expected that by enhancing nurses’ psychological detachment ability and professional mission sense, the impact of inter-nurse horizontal violence on nurses’ job burnout can be reduced, providing strategies for preventing and improving nurses’ job burnout.

## 2 Materials and methods

### 2.1 Study design and participants

From November to December 2024, a cross-sectional design was employed to randomly recruit 580 nurses from five tertiary first-class hospitals in Sichuan, China, for survey analysis. Twenty incomplete responses and twenty-eight unreasonable response patterns were excluded. Ultimately, 532 questionnaires were included in the analysis, with an effective rate of 91.72%.

The inclusion criteria for this study are as follows: registered nurses in active service, with a work experience of ≥1 year, working in the front line of clinical practice, and having provided informed consent and voluntarily cooperating to complete this survey.

The exclusion criteria for this study are as follows: individuals who are not on duty due to reasons such as external studies, training, sick leave, personal leave, maternity leave, as well as retired, intern, or further education nurses.

The sample size was estimated based on the method provided by Kendall (33), which suggests that the sample size should be 5 to 10 times the maximum number of items in a single scale in the study. The maximum variable in a single scale in this study is 22. Considering an invalid questionnaire rate of 10–20%, the required sample size was determined to be between 132 and 264 participants. To ensure the credibility of the research results, as many participants as possible were included in this study, and ultimately 532 participants were included.

### 2.2 Ethical considerations

This study adheres to the ethical standards outlined in the Declaration of Helsinki and has been approved by the Ethics Committee of Deyang People’s Hospital. Prior to the participation of all subjects, informed consent was obtained. The personal information of participants is private and confidential, and is handled anonymously.

### 2.3 Research tools

#### 2.3.1 General information survey form

This questionnaire is designed based on the summary of previous literature research results and expert consultation. It includes factors such as gender, age, educational level, marital status, professional title, work experience, number of night shifts per month, income status, and appointment method.

#### 2.3.2 Horizontal violence among nurses

The “Nurse-to-Nurse Horizontal Violence Questionnaire” designed by Li was used to measure inter-nurse horizontal violence (34). The questionnaire consisted of two dimensions, including eight items on overt violence and eleven items on covert violence. The questionnaire surveyed the frequency of exposure to horizontal violence experienced by the subjects over the past 6 months, using the Likert 5-point scoring system to indicate the frequency of exposure to horizontal violence: 1 point represents “never experienced”; 2 points represent “yes, but rarely experienced”; 3 points represent “almost once a month”; 4 points represent “almost once a week”; 5 points represent “almost once a day.” The total score ranges from 19 to 95, with a higher score indicating more severe horizontal violence. The Cronbach’s *α* coefficient for this questionnaire was 0.953 (34), indicating good reliability and validity. In this study, the Cronbach’s α coefficient was 0.950.

#### 2.3.3 Job burnout scale

The job burnout scale is based on the job burnout-service industry version developed by American scholars Maslach et al. (35). Chinese scholars Feng et al. (36) translated and revised it into its Chinese version, which is more in line with China’s national conditions after revision. It consists of three dimensions and 22 items, including low personal accomplishment (8 items), emotional exhaustion (9 items), and depersonalization (5 items). The Likert 7-point scoring method is adopted, where “never” is scored as 0, “several times a year” as 1, “once a month” as 2, “several times a month” as 3, “once a week” as 4, “several times a week” as 5, and “every day” as 6. In this study, personal accomplishment is scored inversely, and the total score is used to assess the degree of job burnout. The higher the score, the greater the degree of job burnout. The reliability and validity of this scale have been validated in many countries and regions (37). In this study, the Cronbach’s *α* coefficient is 0.890.

#### 2.3.4 Psychological detachment scale

The measurement of psychological detachment level utilizes the psychological detachment scale developed by Sonnentag et al. (38), which was translated and revised into its Chinese version by Lu et al. (39) in consideration of the Chinese cultural background. This scale is a unidimensional scale consisting of four items, utilizing the Likert 5-point scoring method, where “completely disagree” is scored as 1, “somewhat disagree” as 2, “uncertain” as 3, “somewhat agree” as 4, and “completely agree” as 5. The total score ranges from 4 to 20, with a higher score indicating a higher level of psychological detachment in an individual. This scale is widely used and exhibits good reliability and validity, with a Cronbach’s *α* of 0.84 (39). In this study, the Cronbach’s α coefficient is 0.832.

#### 2.3.5 Professional mission scale

The professional calling scale, developed by Zhang et al. (40), consists of 3 dimensions and 10 items, encompassing Guidance (4 items), Altruistic Contribution (3 items), and Initiative (3 items). The scale employs a Likert 5-point scoring system, with “Completely Disagree” scoring 1, “Somewhat Disagree” scoring 2, “Neutral” scoring 3, “Somewhat Agree” scoring 4, and “Completely Agree” scoring 5. The total score ranges from 10 to 50, with a higher score indicating a stronger sense of professional calling. The Cronbach’s *α* coefficient for this scale across groups representing various professions is 0.89 (40). In this study, the Cronbach’s α coefficient for the scale is 0.957, indicating good reliability.

### 2.4 Data collection and quality control methods

With the consent of the nursing managers of the hospitals where the respondents worked, the researchers distributed the questionnaires through their assistance. This survey was conducted using the WeChat-based “Questionnaire Star” platform. The questionnaire included explanations of the research purpose, significance, content, inclusion and exclusion criteria, emphasizing the accuracy and reliability of the survey data. At the same time, the respondents were informed that their relevant information would be strictly confidential and used solely for research purposes. Participants were informed that they could complete the survey voluntarily and anonymously. The first page and each section of the questionnaire included instructions for filling out. Each IP address or WeChat account could only submit once, and the submission time was set to 10 min. The collected questionnaires were carefully reviewed and entered by two researchers, and those with illogical answers, obvious errors, or regular patterns were excluded.

### 2.5 Statistical analyses

Analysis was conducted using SPSS 27.0. General information of the subjects was described using frequency and percentage. After normal distribution testing of the remaining data, the median and quartiles (P25, P75) were used for description. Spearman correlation analysis was employed to examine the correlation between horizontal violence, job burnout, psychological detachment, and professional mission. The Harman’s one-way ANOVA was used to detect common method bias. Model 4 in Hayes’ Process v4.2 macro program was used to verify the mediating effect of psychological detachment between horizontal violence and job burnout. After data de-centralization, regression analysis was conducted to explore the intrinsic relationship between variables. Model 8 in Process v4.2 was used to verify whether professional mission moderated the first half of the mediating effect model and the direct effect. The moderating effect was further analyzed using the simple slope method, and a simple slope plot was drawn using Excel. Both the mediating effect and moderating mediating effect tests used the Bootstrapping method (*n* = 5,000), and a 95% confidence interval was calculated. If the 95% CI did not include zero, it was considered that the mediating effect and moderating mediating effect were valid (41). The test level was set at *α* = 0.05.

## 3 Results

### 3.1 Common method bias test

A common method bias test was conducted on all variables using the Harman’s one-way ANOVA. The results indicated that the characteristic roots of nine factors were greater than 1, and the variance explained by the first factor was 22.91%, which was less than the critical value of 40%. This suggests that there is no significant common method bias in this study.

### 3.2 General information of the surveyed nurses

Ultimately, a total of 532 questionnaires were included for analysis in this study. The general demographic information of the surveyed nurses is detailed in Table 1.

**Table 1:** General demographic information (*n* = 532).

Variables	Category	Number	Percentage(%)
Gender	Male	81	15.23
	Female	451	84.77
Age	Aged 20–30	217	40.79
	Aged 31–40	227	42.67
	Aged 41 and above	88	16.54
Marital status	Not married	112	21.05
	Married	398	74.81
	Divorced	22	4.14
Education level	Junior college	94	17.67
	Bachelor’s	401	75.38
	Master’s	37	6.95
Professional title	Junior nurse	260	48.87
	Intermediate nurse	224	42.11
	Senior nurse	48	9.02
Monthly Income(RMB)	Up to 5,000	50	9.40
	5,001–8,000	298	56.02
	8,001–12,000	108	20.30
	12,001 and above	76	14.29
Years of service	0–5 years	152	28.57
	6–10 years	155	29.14
	11–20 years	150	28.20
	>20 years	75	14.10
Number of night shifts per month	0	118	22.18
	1–5	141	26.50
	6–10	239	44.92
	>10	34	6.40
Employment method	Contract	429	80.64
	Establishment	103	19.36

### 3.3 Correlation analysis between variables

The scores of each variable were described using the median and quartiles, and the correlation among the scales was analyzed using Spearman’s correlation analysis. The results showed that there was a positive correlation between horizontal violence and job burnout (*r = 0.513, p < 0.01*), a negative correlation between horizontal violence and psychological detachment (*r = −0.356, p < 0.01*), a negative correlation between psychological detachment and job burnout (*r = −0.623, p < 0.01*), a positive correlation between professional mission and psychological detachment (*r = 0.361, p < 0.01*), a negative correlation between professional mission and job burnout (*r = −0.355, p < 0.01*), and no significant correlation between professional mission and horizontal violence. Details are shown in Table 2.

**Table 2:** Correlation analysis between variables.

Variable	Horizontal violence	Psychological detachment	Professional mission	Job burnout
Horizontal violence	1	-	-	-
Psychological detachment	−0.356**	1	-	-
Professional mission	−0.041	0.361**	1	-
Job burnout	0.513**	−0.623**	−0.355**	1
M	46.50	9.00	34.00	86.00
(p25, p75)	(44.00,49.00)	(7.00,10.00)	(23.00,44.00)	(77.00,91.00)

**, at the 0.01 level (two-tailed), the correlation is significant.

### 3.4 The mediating effect of psychological detachment

To verify the mediating effect of psychological detachment in the relationship between horizontal violence and job burnout, this study employed Model 4 from the PROCESS v4.2 macro program to construct a mediating effect model. Before data analysis, collinearity detection was conducted. A VIF (Variance Inflation Factor) of ≤ 5 indicates a low risk of collinearity. Taking job burnout as the dependent variable and horizontal violence and psychological detachment as independent variables, the measured VIF values were both 1.055; taking psychological detachment as the dependent variable and horizontal violence as the independent variable, the measured VIF value was 1.000. This indicates no significant collinearity between the variables. The Bootstrap sampling method (sample size = 5,000, confidence level 95%) was used to test the indirect effect. If the 95% confidence interval (CI) does not include 0, then the mediating effect is significant. The results, as presented in Table 3, indicate that horizontal violence significantly predicts job burnout (*β = 0.151, p < 0.001*) and also significantly negatively predicts psychological detachment (*β = −0.229, p < 0.001*). Conversely, psychological detachment significantly negatively predicts job burnout (*β = −0.533, p < 0.001*). Upon testing the mediating model, the results, as shown in Table 4, reveal that horizontal violence has a significant direct effect on job burnout (*B = 0.203, p < 0.001*), with a 95% confidence interval (CI) of (*0.107–0.299*). Additionally, the indirect effect via psychological detachment is also significant (*B = 0.164, p < 0.001*), with a 95% CI of (*0.052–0.267*). The mediating effect accounts for 44.73% of the total effect.

**Table 3:** Regression results of the mediating effect of psychological detachment.

Dependent variable	Independent variable	*β*	*SE*	*t*	*P*	*R^2^*	*F*
Psychological detachment	Horizontal violence	−0.229	0.011	−5.408	0.000	0.052	29.241
Job burnout	Horizontal violence	0.151	0.049	4.165	0.000	0.344	138.715
	Psychological detachment	−0.533	0.180	−14.747	0.000		

**Table 4:** The mediating effect of psychological detachment between inter-nurse horizontal violence and job burnout.

Project	Effect value	SE	95%CI	*p*	Relative effect value (%)
Direct effect	0.203	0.049	0.107–0.299	0.000	55.27%
Indirect effect	0.164	0.056	0.052–0.267	0.000	44.73%
Total effect	0.368	0.056	0.257–0.478	0.000	100.00%

### 3.5 The moderating effect of professional mission

To verify the moderating effect of professional mission in the mediation model, this study conducted regression analysis on each variable after de-centering (subtracting their respective means), and the results are shown in Table 5. Professional mission can significantly moderate the impact of horizontal violence on job burnout (*B = −0.032, p < 0.001*) and can also significantly moderate the impact of horizontal violence on psychological detachment (*B = 0.009, p < 0.001*). To further verify the hypothesis, Model 8 in the PROCESS v4.2 macro program was used to construct and validate a moderating effect model. The results are shown in Table 6. As professional mission increases, the mediating effect value of psychological detachment in the relationship between horizontal violence and job burnout gradually increases. The mediating effect remains significant under low and medium levels of professional mission, with 95% CIs not crossing 0. However, under high levels of professional mission, the mediating effect model is no longer significant.

**Table 5:** Analysis and examination of the moderating effect of professional mission.

Variable	Equation 1			Equation 2			Equation 3		
	*B*	*β*	*t*	*B*	*β*	*t*	*B*	*β*	*t*
Horizontal violence	0.360	0.051	7.084 ***	−0.061	0.010	−6.243 *	0.231	0.048	4.793 ***
Professional mission	−0.377	0.043	−8.682 ***	0.082	0.008	9.910 ***	−0.201	0.043	−4.664 ***
Horizontal violence × Professional mission	−0.032	0.004	−7.282***	0.009	0.001	10.963***	−0.012	0.004	−2.778 **
Psychological detachment	-	-	-	-	-	-	−2.135	0.208	−10.246***
*R^2^*	0.249			0.321			0.374		
*F*	58.426***			83.263***			78.692***		

Equation 1: dependent variable: job burnout. Equation 2: dependent variable: psychological detachment. Equation 3: dependent variable: job burnout. *p < 0.05, **p < 0.01, ***p < 0.001.

**Table 6:** The impact of different levels of adjustment on the mediation effect size in the mediation model.

Level of professional mission	Mediating effect size	BootSE	BootLLCI	BootULCI
Low	0.356	0.061	0.253	0.491
Medium	0.129	0.038	0.066	0.215
High	−0.097	0.061	−0.201	0.034

Low: M-SD; Medium: Mean; High: M + SD.

**Figure 2:**
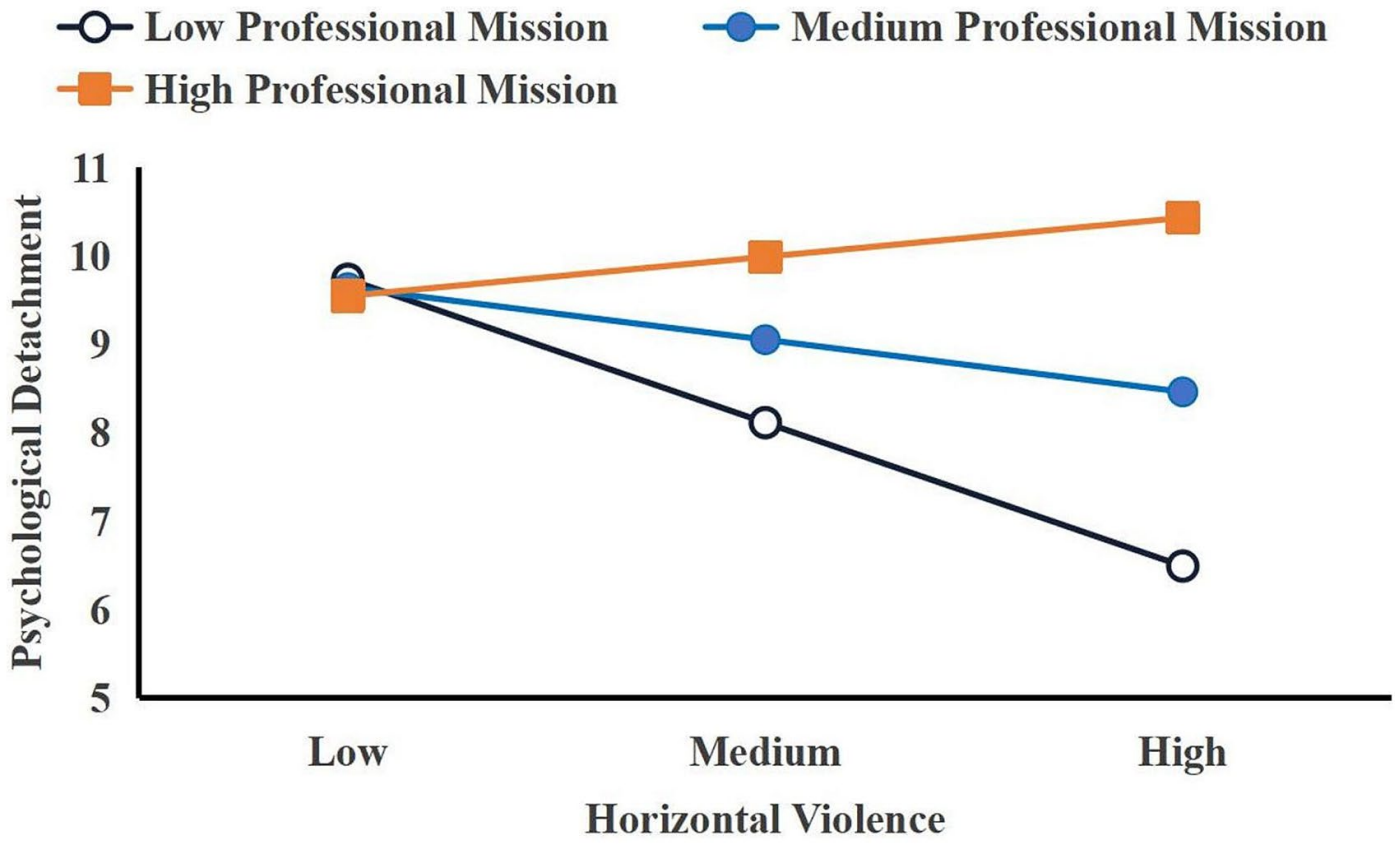
Simple slope diagram of the moderating effect of professional mission on the relationship between horizontal violence and psychological detachment.

To further visually observe the moderating effect of professional mission on the first half of the mediation effect model and the direct effect, a simple slope test was further used for analysis, with professional mission categorized into low, medium, and high levels, corresponding to M-SD, M, and M + SD, respectively. As shown in Figure 2, when professional mission is at a low level, horizontal violence can significantly negatively predict psychological detachment (*βsimple = −0.167, p < 0.001*). When professional mission is at a medium level, horizontal violence can still significantly negatively predict psychological detachment (*βsimple = −0.061, p < 0.001*). When professional mission is at a high level, horizontal violence can still positively predict psychological detachment (*βsimple = 0.045, p < 0.001*). This indicates that as professional mission increases, the negative prediction of horizontal violence on psychological detachment gradually weakens, suggesting that professional mission plays a positive moderating role in the impact of horizontal violence on psychological detachment.

**Figure 3:**
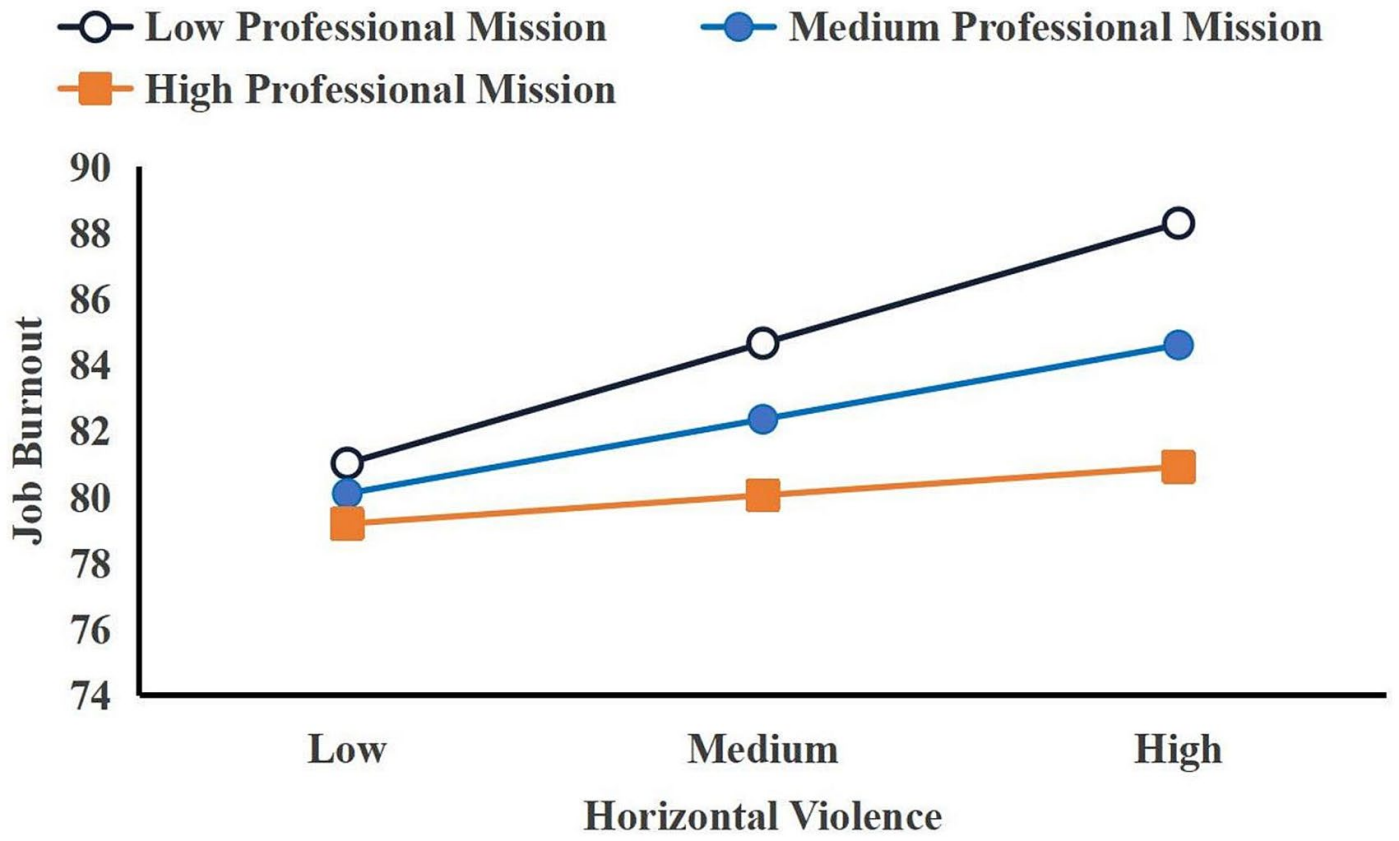
Simple slope diagram of the moderating effect of professional mission on the relationship between horizontal violence and job burnout.

As shown in Figure 3, when professional mission is at a low level, horizontal violence can significantly positively predict job burnout (βsimple = 0.373, *p* < 0.001). When professional mission is at a medium level, horizontal violence can still significantly positively predict job burnout (*βsimple = 0.231, p < 0.001*). However, when professional mission is at a high level, horizontal violence cannot effectively predict job burnout (*βsimple = 0.089, p = 0.180*). Furthermore, as professional mission increases, the positive prediction of horizontal violence on job burnout gradually weakens, indicating that professional mission plays a negative moderating role in the impact of horizontal violence on job burnout.

## 4 Discussion

### 4.1 The impact of horizontal violence among nurses on job burnout

Due to the unique social environment in China, nurses have been working under high-intensity conditions for a long time, making them highly susceptible to horizontal violence. In this study, the score for horizontal violence among nurses was above the medium level, indicating that the problem of horizontal violence among Chinese nurses is relatively prominent. Castronovo et al. (42) found that horizontal violence has a negative impact on nurses’ physical and mental health, leading to varying degrees of anxiety, depression, fatigue, insomnia, decreased self-confidence, and impaired self-esteem. Prolonged exposure to such distress and pressure may even pose a suicide risk. According to the COR (13) theory and the JD-R (15) model, experiencing horizontal violence depletes nurses’ “emotional resources.” When faced with high-intensity work demands, insufficient work resources can lead to errors, decreased work efficiency, and job burnout among nurses. Farrell et al. (43) confirmed this viewpoint, finding that two-thirds of nurses who experienced horizontal violence made errors or accidents in nursing work, exhibited negative work emotions, and developed job burnout, subsequently leading to a decline in nursing quality. In this study, the score for nurses’ job burnout was relatively high, indicating that nursing managers need to pay attention to nurses’ job burnout and actively intervene. This study also found a significant positive correlation between horizontal violence and nurses’ job burnout, meaning that as horizontal violence increases, the level of nurses’ job burnout also increases, confirming Hypothesis 1 and aligning with previous research findings (16). Renata et al. (44) further found that newly hired nurses are more prone to experiencing horizontal violence from experienced nurses. The analysis pointed out that job burnout among senior colleagues is one of the reasons for newly hired colleagues experiencing horizontal violence. Therefore, there may be a mutual causality between horizontal violence and job burnout, which can be further explored in future research.

It can be inferred that the depletion of “emotional resources” and the imbalance between job resources and job demands may be potential causes of job burnout among nurses after experiencing horizontal violence. To reduce the occurrence of horizontal violence among nurses and job burnout, it is recommended that nursing managers start with the following aspects. Firstly, increase departmental team-building activities to enhance the relationship between nurses and strengthen team collaboration. Secondly, implement more reasonable staffing arrangements to reduce nurses’ work intensity and allow them sufficient time to rest and recover their “job resources.” Thirdly, provide more care for nurses’ lives and work, communicate more, pay attention to nurses’ psychological changes, and detect and intervene early.

### 4.2 The mediating role of psychological detachment

The results of this study indicate that psychological detachment is significantly negatively correlated with both interpersonal horizontal violence and job burnout, confirming Hypotheses 2 and 3. Psychological detachment plays a partial mediating role in the relationship between interpersonal horizontal violence and job burnout, supporting Hypothesis 4. According to the Effort-Recovery Theory (21) and the COR (13) theory, nurses who experience interpersonal horizontal violence need timely recovery of their “emotional resources” to be able to handle subsequent high-intensity work. Related research shows (20) that psychological detachment, as a means of restoring individual psychological resources, can help nurses recover the “emotional resources” depleted during work. During psychological detachment, nurses may engage in activities unrelated to work, such as leisure, enjoying food, sports, and travel, thereby acquiring new “emotional resources” to better engage in their work.

Therefore, nurses with higher psychological detachment ability can fully disconnect from work mentally during their break time. This psychological “state of mindfulness” allows nurses to stop consuming their personal “emotional resources,” and nurses with higher psychological detachment ability also have a stronger ability to recover their “emotional resources” during breaks (22, 45). This is beneficial for reducing the damage of horizontal violence to nurses’ physical and mental health and alleviating the sense of professional burnout brought about by high-intensity work. Conversely, nurses with lower psychological detachment ability still recall the horizontal violence they experienced at work during non-work periods, leading to continuous consumption of “emotional resources” during break time. Long-term emotional consumption leads to the occurrence of professional burnout (23).

The results of this study indicate that improving nurses’ psychological detachment ability can effectively block nearly 45% of the transmission pathway from horizontal violence to job burnout, providing specific targets for clinical intervention. It is recommended that nursing managers arrange work reasonably, establish a good communication mechanism, and avoid disturbing nurses’ rest during non-working hours. If it is indeed impossible to reduce nurses’ workload and working hours, it is suggested that nursing managers adopt a positive psychology perspective, cultivate nurses’ psychological detachment ability, and encourage nurses to develop their own hobbies and participate in sports activities. This will enable nurses to achieve effective psychological detachment during their rest time, thereby restoring their physical and mental resources and allowing them to have sufficient working resources to apply to their work, thus alleviating job burnout.

### 4.3 The moderating effect of professional mission

The results of this study indicate that there is a significant negative correlation between professional mission, psychological detachment, and job burnout, confirming Hypotheses 5 and 6. Professional mission moderates both the first half of the mediation effect model and the direct effect. As professional mission increases, the mediation effect value of the mediation model gradually decreases, confirming Hypothesis 7. Further simple slope analysis reveals that professional mission negatively moderates the negative prediction of horizontal violence on psychological detachment and simultaneously negatively moderates the positive prediction of horizontal violence on job burnout. Specifically, according to MMT (28) theory, nurses with a strong sense of professional mission can, through cognitive restructuring, perceive the horizontal violence they face as a test of self-worth, thereby enhancing their psychological detachment and psychological resilience, ultimately reducing their job burnout.

The bootstrap method revealed that when nurses possess a strong sense of professional mission, this sense does not effectively moderate the direct effect within the mediation model. Additionally, the 95% confidence interval (CI) of the mediation effect model includes zero, indicating that the mediation effect is not significant. The potential reason for this is that nurses with a strong sense of professional mission may deeply recognize the value and significance of nursing work, thus cherishing and loving their profession more, consciously taking responsibility, and firmly committing to their nursing work (31, 46). Therefore, when they encounter horizontal violence, their “emotional resources” are rarely depleted, making active psychological detachment unnecessary for restoring their psychological resources.

Therefore, it is recommended that nursing managers, while focusing on how to enhance nurses’ psychological detachment, also consider how to boost their sense of professional mission. Nursing managers can help nurses maintain a positive learning attitude, enhance their core competencies, and become more competent in their work by assisting them in adhering to reasonable professional norms, enabling them to gain a sense of achievement in their work (47). Secondly, a scientific, fair, and long-term performance distribution system should be established to ensure that nurses receive income commensurate with their work intensity, reflecting the value of their labor (48). In addition, nursing managers should fully leverage their organizational coordination role, optimize human resource management models, and reduce negative events that affect nurses’ job satisfaction, such as horizontal violence among nurses (49). The above measures can increase nurses’ sense of professional mission to a certain extent, thereby reducing the impact of horizontal violence on job burnout.

## 5 Conclusion

The results of this study indicate that Chinese nurses face relatively severe issues of horizontal violence and job burnout. Inter-nurse horizontal violence can significantly positively predict job burnout. Psychological detachment plays a partial mediating role in the relationship between horizontal violence and job burnout. Professional mission moderates the first half of the mediation effect model and the direct effect. Specifically, professional mission negatively moderates the negative prediction of horizontal violence on psychological detachment and negatively moderates the positive prediction of horizontal violence on job burnout.

In summary, this study indicates that enhancing nurses’ psychological detachment and sense of professional mission can significantly mitigate the impact of inter-nurse horizontal violence on job burnout. It is recommended that nursing managers employ effective methods to improve nurses’ psychological detachment and sense of professional mission, which will contribute to enhancing nurses’ physical and mental health, while also improving the quality of nursing care.

## 6 Study limitations

This study verifies the mediating effect of psychological detachment between inter-nurse horizontal violence and job burnout, and further verifies the moderating effect of professional mission in the mediating effect model. This study has the following limitations. Firstly, this study only included 532 nurses from the southwest region of China, which is a relatively small sample size. Due to factors such as regional economy, culture, and population, the results are somewhat limited. Future research with more regions and larger sample sizes is needed for further verification, in order to draw more extensive and applicable conclusions. Secondly, the questionnaires and scales used in this study are subjective in answering, which may lead to consistency bias. Although quality control measures were employed in this study, methodological bias cannot be completely avoided. Thirdly, this study is a cross-sectional survey, reflecting only the situation at that time and unable to clarify the changes in relationships between variables based on temporal changes. In the future, longitudinal design can be further adopted to clarify the dynamic relationships between variables.

## Data Availability

The original contributions presented in the study are included in the article/Supplementary material, further inquiries can be directed to the corresponding author.
